# Pain and functional outcomes after surgical versus hormonal treatment in rectovaginal endometriosis: a retrospective cohort study

**DOI:** 10.1007/s00404-026-08495-z

**Published:** 2026-06-21

**Authors:** Franziska Werner, Renata Voltolini Velho, Sylvia Mechsner

**Affiliations:** Department of Gynaecology Charité with Centre of Oncological Surgery, Endometriosis Research Centre Charité, Campus Virchow-Klinikum, Augustenburger Platz 1, 13353 Berlin, Germany

**Keywords:** Endometriosis, Deep-infiltrating endometriosis, Rectovaginal endometriosis, Surgical treatment, Hormonal therapy, Pelvic pain

## Abstract

**Purpose:**

To compare changes in pain-related symptoms, bowel and bladder function, rectal bleeding, quality of life, and treatment satisfaction in women with rectovaginal endometriosis treated either surgically or with hormonal treatment alone in a tertiary referral centre.

**Methods:**

This retrospective cohort study included women with rectovaginal endometriosis treated at a tertiary endometriosis centre with either surgical excision or hormonal treatment alone after informed consent. Standardised questionnaires assessed pain, functional symptoms, quality of life, and treatment satisfaction. Symptom changes were categorised as improvement, stability, or worsening. Between-group comparisons were performed using Mann–Whitney U tests and Pearson’s chi-square tests, with effect sizes reported (r or Cramér’s V). The analysis was exploratory.

**Results:**

A total of 210 women were included (surgical *n* = 164; hormonal *n* = 46). Baseline pain intensity did not differ significantly between groups, although bowel dysfunction and rectal bleeding were more prevalent in the surgical cohort. Following treatment, approximately 80% of women in both groups reported improvement in pelvic pain and dysmenorrhoea. Improvements in dyspareunia, dyschezia, and functional outcomes were observed in substantial proportions. Between-group comparisons revealed no statistically significant differences in change categories across pain, functional symptoms, quality of life, or treatment satisfaction (all *p* ≥ 0.08), with consistently small effect sizes.

**Conclusion:**

Both surgical and hormonal treatment were associated with substantial improvements in patient-reported outcomes in women with rectovaginal endometriosis. Direct comparison revealed no significant differences in outcome trajectories, supporting an individualised treatment approach.

**Supplementary Information:**

The online version contains supplementary material available at 10.1007/s00404-026-08495-z.

## What does this study add to the clinical work

**Table:** 

Our findings suggest that, in the absence of strict surgical indications, hormonal therapy may be a viable treatment option worthy of further investigation for women with rectovaginal endometriosis. These data may assist clinicians in counselling patients by providing realistic expectations regarding symptom improvement and treatment satisfaction with both strategies.	

## Introduction

Endometriosis is an oestrogen-dependent, chronic inflammatory condition affecting up to 20% of women of reproductive age worldwide. It is characterised by the presence of endometrium-like tissue outside the uterine cavity and manifests as superficial peritoneal lesions, ovarian endometriomas, or deep-infiltrating endometriosis (DIE) [9, 15]. Clinical presentation is heterogeneous and commonly includes dysmenorrhoea, chronic pelvic pain, dyspareunia, dyschezia, and dysuria, often accompanied by functional bowel and bladder symptoms [17, 25]. The resulting symptom burden can substantially impair health-related quality of life and psychosocial functioning [4, 21].

Rectovaginal endometriosis (RVE) represents one of the most severe phenotypes of DIE. Lesions typically infiltrate the rectovaginal septum and frequently involve the posterior vaginal wall and rectum. Due to fibrotic infiltration and proximity to pelvic nerves, RVE is frequently associated with severe pain symptoms, including deep dyspareunia and dyschezia, and may contribute to bowel dysfunction and rectal bleeding. The complex anatomical involvement and potential neuropathic pain components make management particularly challenging [7, 18].

Treatment strategies for RVE include both surgical and medical approaches. Surgical management aims at complete excision of endometriotic lesions and may result in substantial symptom relief. However, surgery carries perioperative risks and may be associated with long-term complications, particularly bowel and bladder dysfunction [22, 30]. Hormonal treatment, including progestins or combined hormonal contraceptives, is widely used as a first-line treatment for endometriosis-associated pain [2, 27]. By suppressing ovulation and reducing inflammatory activity, hormonal treatment may decrease nociceptive input and symptom severity. Nevertheless, side effects and limited suitability for women actively seeking pregnancy restrict its use in some patients. [11, 18].

While surgical outcomes in DIE have been extensively studied [7, 12, 30], and hormonal treatment is well established for pain control in endometriosis in general [26], direct comparative data specifically addressing patient-reported outcomes after surgical versus hormonal management in RVE remain limited.

The aim of this retrospective cohort study was therefore to compare changes in pain-related symptoms, bowel and bladder function, rectal bleeding, quality of life, and treatment satisfaction in women with RVE treated either surgically or with hormonal treatment alone in a tertiary referral centre. By directly contrasting these management strategies, this study seeks to provide clinically relevant data to support individualised treatment decisions in this complex pain condition.

## Methods

### Study design

This retrospective cohort study was conducted at a certified tertiary endometriosis centre between 2009 and 2018. Eligible patients were contacted and invited to complete a study-specific questionnaire, which is available in the Supplementary Material 1.

Participants retrospectively reported symptom severity prior to treatment and at follow-up. Pain intensity (pelvic pain, dysmenorrhoea, dyspareunia, dyschezia) was assessed using an 11-point numeric rating scale (NRS) from 0 to 10; a rating of 0 indicates the absence of pain, whereas a rating of 10 indicates the strongest imaginable pain. For analytical purposes, these scores were treated as ordinal variables.

The presence of bowel and bladder dysfunction, rectal bleeding, quality of life, and treatment satisfaction were assessed as dichotomous variables.

For comparative analyses, change variables were generated for each symptom and categorised as improved, unchanged, or worsened.

The primary outcome was the comparison of changes in pain symptoms (pelvic pain, dysmenorrhoea, dyspareunia, dyschezia) between the surgical and hormonal group. Secondary outcomes included changes in bowel and bladder function, rectal bleeding, quality of life, and treatment satisfaction between both groups.

### Study population

Women were eligible if they (i) were between 18 and 60 years of age, (ii) had a confirmed diagnosis of RVE, (iii) had undergone either surgical treatment of RVE or hormonal treatment only, and (iv) consented to participation by returning the questionnaire. All included patients had been treated at the Endometriosis Centre Charité-Universitätsmedizin Berlin during the study period.

The choice of treatment was based on shared decision-making between patient and treating physician, considering symptom severity, lesion extent, reproductive plans, and patient preference. For the analysis, patients were assigned to one of two groups based on the treatment strategy:

Hormonal treatment group: patients who received non-surgical, hormonal treatment only, including gestagens, combined oral contraceptives, GnRH analogues/antagonists, and/or other medical therapies, alone or in combination.

Surgical group: patients who underwent laparoscopic or open surgical excision of RVE. In 63 of these women, bowel resection was performed in addition to excision of rectovaginal lesions.

### Statistical analysis

Statistical analyses were performed using IBM SPSS Statistics (version 31.0.0.0). Missing responses were not replaced.

First, it was examined whether the two treatment groups differed in baseline symptoms before treatment. Ordinal variables, including pain intensity scores, are presented as medians with interquartile range (IQR) and compared using exact Mann–Whitney U tests. Categorical variables, including presence of functional symptoms and quality of life, are reported as absolute and relative frequencies and compared using Pearson’s chi-square tests.

For longitudinal evaluation, change variables were generated for each symptom and quality of life categorised as improved, unchanged, or worsened based on the direction of change between pre- and post-treatment.

To compare these change variables between the surgical and hormonal group, change variables were analysed using the Mann–Whitney U test. Treatment satisfaction between both groups was compared using Pearson’s chi-square test.

Effect sizes were calculated as r for Mann–Whitney U tests and Cramér’s V for chi-square tests, with interpretation according to Cohen’s conventional cut-offs (|0.1–0.3| small, |0.3–0.5| medium, >|0.5| large). All tests were two-sided. Given the exploratory nature of the study, no formal correction for multiple testing was applied; all findings should, therefore, be considered hypothesis-generating and interpreted with caution given the increased risk of type I error. A *p *value < 0.05 was considered statistically significant [3].

## Results

### Baseline characteristics

A total of 210 women with RVE returned evaluable questionnaires and were included in the analysis. Of these, 164 (78.1%) had undergone surgical treatment and 46 (21.9%) had received hormonal treatment only.

At baseline, women in both groups reported substantial symptom burden. Median NRS scores for pelvic pain were 7 [IQR 4] in the surgical group and 6 [IQR 4] in the hormonal treatment group, with no significant between-group difference (*p* = 0.370, *r* = − 0.065). Baseline NRS scores for dysmenorrhoea, dyspareunia, and dyschezia showed no statistically significant differences between groups (all *p* > 0.05), although dyschezia scores were numerically higher in the surgical group.

Bladder dysfunction and impaired quality of life were common in both groups and did not differ significantly at baseline. In contrast, rectal bleeding (29.1% vs. 11.4%, *p* = 0.017, *V* = 0.169) and bowel dysfunction (68.5% vs. 45.5%, *p* = 0.005, *V* = 0.196) were significantly more frequently reported in women who subsequently underwent surgery, indicating a higher baseline burden of bowel-related symptoms in this group.

With approximately 90% of respondents in both treatment groups, a clear majority of respondents stated that their quality of life was impaired prior to therapy. Baseline characteristics and symptoms are summarised in Table 1.

**Table 1:** Baseline pain and functional symptoms in women with rectovaginal endometriosis, by treatment group

	NRS Median [IQR]	n (Percentage)	*p*	*r*/*V*
Pelvic pain			0.370	− 0.065
Surgical	7 [4]	142 (86.6)		
Hormonal	6 [4]	36 (78.3)		
Dysmenorrhoea			0.849	− 0.014
Surgical	7 [4]	133 (81.1)		
Hormonal	7 [3]	33 (71.7)		
Dyspareunia			0.983	− 0.002
Surgical	3 [6]	134 (81.7)		
Hormonal	3 [5]	32 (69.6)		
Dyschezia			0.151	− 0.104
Surgical	4 [7]	136 (82.9)		
Hormonal	0 [6]	34 (73.9)		
Rectal bleeding			0.017	0.169
Surgical		46 (29.1)		
Hormonal		5 (11.4)		
Bowel dysfunction			0.005	0.196
Surgical		111 (68.5)		
Hormonal		20 (45.5)		
Bladder dysfunction			0.764	0.021
Surgical		37 (22.8)		
Hormonal		11 (25.0)		
Impaired quality of life			0.585	0.039
Surgical		140 (90.9)		
Hormonal		37 (88.1)		

Data are presented as median [IQR] for NRS scores, number (percentage) and *p *values for comparisons between surgical and hormonal group and effect sizes (r or Cramér’s V). NRS, numeric rating scale; IQR, interquartile range; V, Cramér’s V

### Change in pain and functional symptoms

**Fig. 1:**
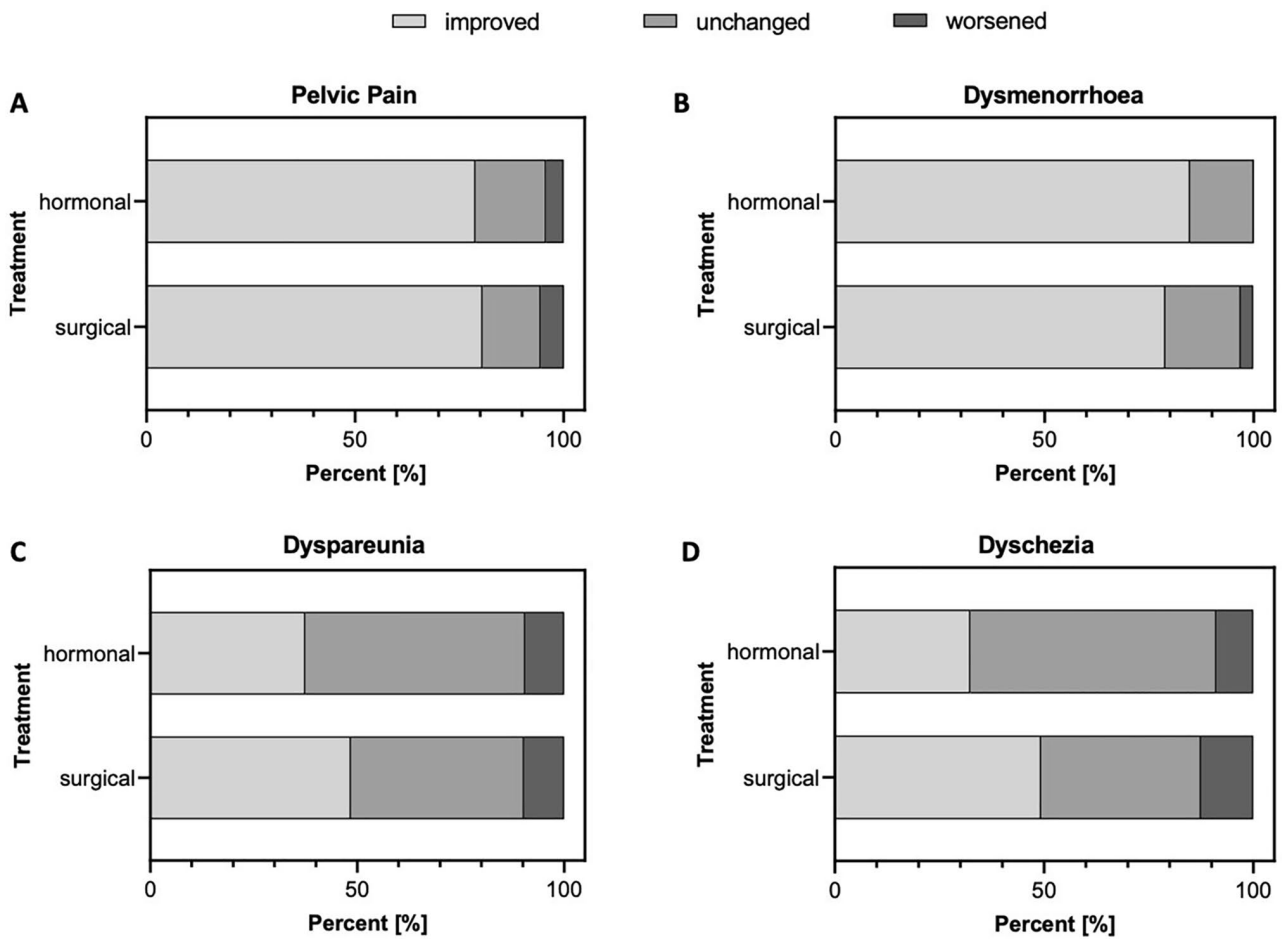
Change in pain outcomes after surgical versus hormonal treatment in women with rectovaginal endometriosis. Stacked bar plots show the proportions of patients reporting improvement, no change, or worsening for. **A** Pelvic pain, **B** dysmenorrhoea, **C** dyspareunia, and **D** dyschezia

Across all pain domains, both treatment strategies were associated with substantial symptom improvement. The distribution of change categories (improvement, no change, worsening) did not differ significantly between surgical and hormonal treatment for pelvic pain, dysmenorrhoea, dyspareunia, or dyschezia (all *p≥*0.211; Table 2, Fig. 1).

**Table 2:** Change in pain, functional outcomes, quality of life, and treatment satisfaction after surgical versus hormonal treatment in women with rectovaginal endometriosis

Therapeutic outcome	Surgical n (percentage)	Hormonal n (percentage)	p	*r*/*V*
Pelvic pain	142	36	0.897	− 0.013
Improved	112 (78.9)	29 (80.6)		
Unchanged	24 (16.9)	5 (13.9)		
Worsened	6 (4.2)	2 (5.6)		
Dysmenorrhoea	133	33	0.411	− 0.063
Improved	105 (78.9)	28 (84.8)		
Unchanged	24 (18.0)	5 (15.2)		
Worsened	4 (3.0)	0 (0.0)		
Dyspareunia	134	32	0.370	− 0.074
Improved	65 (48.5)	12 (37.5)		
Unchanged	56 (41.8)	17 (53.1)		
Worsened	13 (9.7)	3 (9.4)		
Dyschezia	136	34	0.211	− 0.098
Improved	67 (49.3)	11 (32.4)		
Unchanged	52 (38.2)	20 (58.8)		
Worsened	17 (12.5)	3 (8.8)		
Rectal bleeding	146	41	0.080	− 0.138
Improved	32 (21.9)	4 (9.8)		
Unchanged	113 (77.4)	36 (87.8)		
Worsened	1 (0.7)	1 (2.4)		
Bowel dysfunction	149	43	0.142	− 0.110
Improved	28 (18.8)	4 (9.3)		
Unchanged	109 (73.2)	34 (79.1)		
Worsened	12 (8.1)	5 (11.6)		
Bladder dysfunction	150	41	0.708	− 0.072
Improved	14 (9.3)	2 (4.9)		
Unchanged	125 (83.3)	37 (90.2)		
Worsened	11 (7.3)	2 (4.9)		
Quality of life	154	42	0.380	− 0.065
Improved	112 (72.7)	27(64.3)		
Unchanged	37 (24.0)	15 (35.7)		
Worsened	5 (3.2)	0 (0.0)		
Treatment satisfaction	152	40	0.063	0.134
Yes	135 (88.8)	31 (77.5)		
No	17 (11.2)	9 (22.5)		

Data are shown as number (percentage) of patients in each category, *p* values for comparisons between surgical and hormonal group and effect sizes (r or Cramér’s V). V, Cramér’s V

Approximately four out of five women in both groups reported improvement in pelvic pain and dysmenorrhoea. For dyspareunia and dyschezia, improvement rates were lower and a considerable proportion of women reported stable symptoms, particularly in the hormonal treatment group. Worsening of pain symptoms was uncommon in both groups across all domains.

**Fig. 2:**
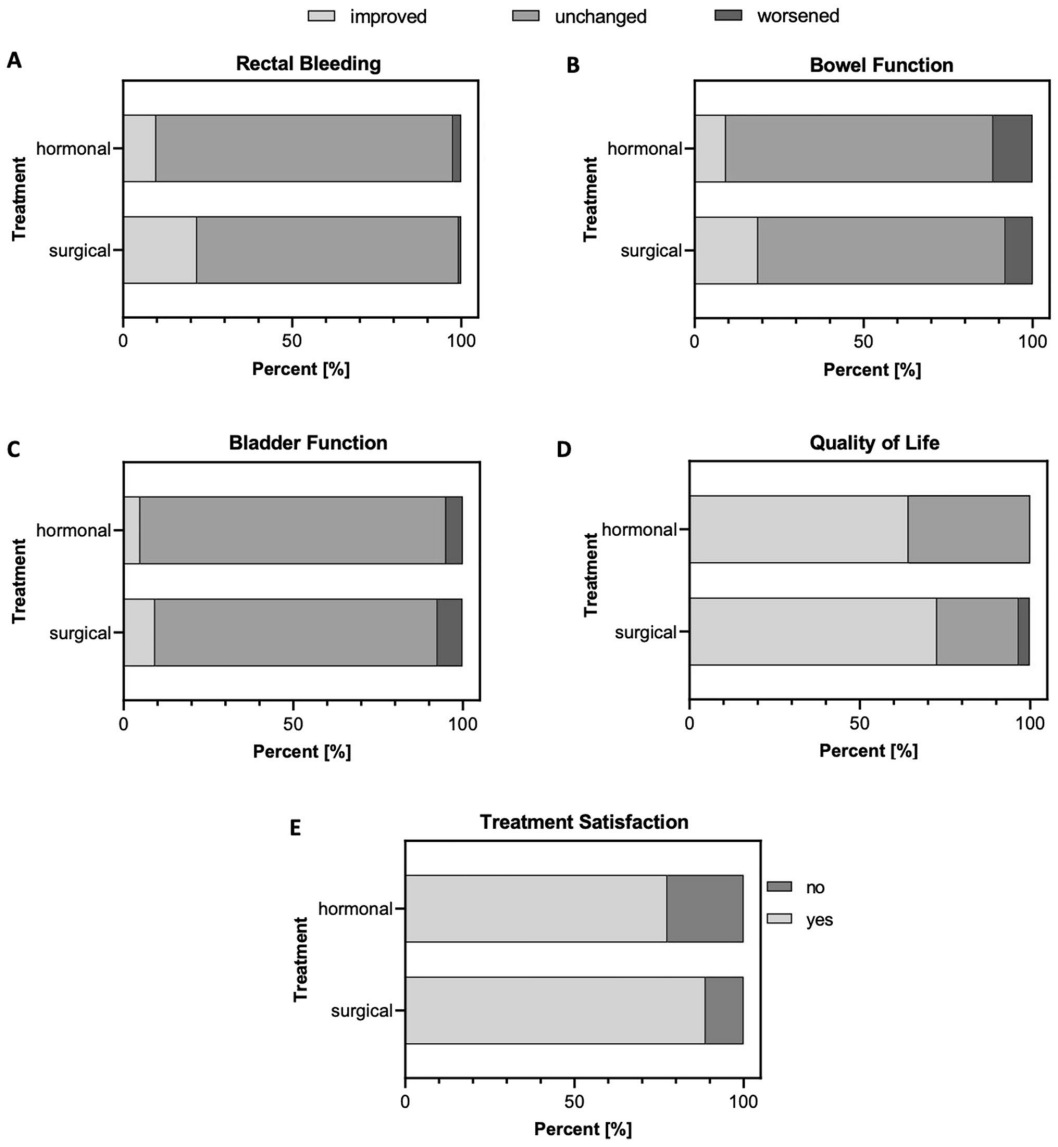
Change in functional outcomes, quality of life and treatment satisfaction after surgical versus hormonal treatment. Stacked bar plots show the proportions of patients reporting improvement, no change or worsening for **A** rectal bleeding, **B** bowel function, **C** bladder function, **D** quality of life, and **E** treatment satisfaction

Similarly, no statistically significant between-group differences were observed for changes in rectal bleeding, bowel dysfunction, or bladder dysfunction (all *p≥*0.08; Table 2, Fig. 2). Most women reported stable functional symptoms, with smaller proportions experiencing improvement and only a minority reporting worsening. Effect sizes for all between-group comparisons were small, indicating minimal differences in outcome patterns between surgical and hormonal management.

### Quality of life and treatment satisfaction

Quality of life improved in the majority of women in both groups with no significant difference in the distribution of change categories between surgical and hormonal treatment (*p* = 0.380; *r* = − 0.065). Worsening of quality of life was rare.

Overall treatment satisfaction was high in both groups. Although satisfaction was numerically higher after surgery (88.8% vs. 77.5%), this difference did not reach statistical significance (*p* = 0.063, *V* = 0.134) and the effect size was small (Table 2, Fig. 2).

## Discussion

In this retrospective cohort study of women with RVE, we observed substantial improvements in pain-related symptoms, functional outcomes, and quality of life following both surgical and hormonal treatment. These findings should not be interpreted as evidence of comparable effectiveness, given the non-randomised design, the smaller hormonal cohort, and the absence of formal equivalence or non-inferiority testing. The results are best regarded as hypothesis-generating, and residual confounding cannot be excluded.

Surgical excision of DIE is widely regarded as the standard treatment for RVE, particularly in patients with bowel involvement. Previous surgical series have reported improvement of 60–100% in dysmenorrhoea, dyspareunia, and chronic pelvic pain [30]. The improvement rates observed in our surgical cohort, approximately 80% in dysmenorrhoea and pelvic pain and almost 50% in dyspareunia, fall within this reported range, indicating that our data fall within the range of established surgical outcome literature.

Evidence for hormonal treatment specifically in RVE is less abundant and only one randomised-controlled trial was identified in the literature [29]. Reported response rates for therapy with 2.5 mg norethindrone acetate per day in endometriosis-associated pain range between 74 and 92%. Although in this cohort, the following pain symptoms were already absent before treatment: 37% for dyspareunia, 20% for non-menstrual pain, and 45% for dyschezia.

A contemporary meta-analysis by Wu et al. evaluating the efficacy and safety of dienogest in women with DIE demonstrated improved pain outcomes and rectosigmoid nodule size, although with increased headache rates and decreased libido [31]. These findings underscore both the potential even in anatomically advanced stages and the characteristic side-effect profile of progestin therapy.

In this context, the symptom improvements observed in our hormonal treatment group are plausible and consistent with broader literature on medical management of DIE.

The pathophysiology of pain in RVE is multifactorial and not determined solely by anatomical involvement. Surgical excision removes macroscopic endometriotic lesions and may relieve local inflammatory foci, mechanical tension and potential nerve entrapment. However, lesion removal does not necessarily eliminate the biological processes that sustain pain [8, 16, 24, 26].

Hormonal therapy, in contrast, does not remove lesions but suppresses ovarian activity and reduces cyclical hormonal stimulation of ectopic endometrial tissue, thereby decreasing inflammatory signalling and nociceptive input [6, 20, 26].

These different mechanisms may converge on similar patient-reported outcomes, even though the biological routes are distinct. This mechanistic perspective is compatible with the observed symptom improvements in both groups but should not be interpreted as proof that the two strategies are clinically equivalent.

We observed a significantly higher prevalence of rectal bleeding and bowel dysfunction in the surgical group at baseline, suggesting that patients with more pronounced bowel involvement were preferentially selected for operative management. This reflects real-world clinical decision-making in tertiary centres [5, 10]. However, residual confounding by indication is a key limitation of this study. Because patients with more severe bowel involvement were preferentially selected for surgery, the observed similarities in outcome trajectories may partly reflect regression to the mean or unmeasured prognostic differences, rather than true treatment equivalence. The absence of adjusted analyses (e.g., multivariable models or propensity score methods) limits causal interpretation. These findings should, therefore, be considered hypothesis-generating only, and prospective controlled analyses are required to confirm whether hormonal treatment provides meaningful symptom relief in patients with more pronounced bowel-related complaints.

From a clinical perspective, our findings support a more individualised treatment approach in RVE. While surgery remains indispensable in selected cases, particularly in the presence of obstructive symptoms or fertility considerations, hormonal therapy appears capable of achieving improvements in patient-reported outcomes in many patients [2, 27]. These results may help facilitate shared decision-making by providing realistic expectations regarding symptom trajectories under both strategies.

The study by Vercellini et al. on colorectal endometriosis provides an important clinical context for our findings. In that work, women who were counselled through a structured shared decision-making process achieved meaningful symptom relief with either surgery or medical treatment, underscoring the importance of patient preferences, expectations, and risk tolerance [28]. Our results are consistent with this framework: in women with RVE, symptom trajectories following the two strategies were broadly favourable, and the choice of treatment should, therefore, be individualised rather than based on assumed universal superiority of surgery.

The high burden of symptoms, which has a significant impact on the overall well-being and mental health of endometriosis patients, requires a therapeutic approach that focuses on improving their quality of life [1]. In consistency with the current literature, our study demonstrates that medical and surgical treatment of (deep-infiltrating) endometriosis may improve health-related quality of life [13, 23, 32]. Although overall quality of life improved in the majority of women in both groups, a small proportion of surgically treated patients reported worsening, which may reflect the potential impact of perioperative or postoperative complications, persistent symptoms, or altered functional outcomes following extensive pelvic surgery. Given the anatomical complexity of RVE, even infrequent adverse sequelae may substantially affect perceived well-being in individual cases [12, 22].

Treatment satisfaction was numerically high after surgery, but this difference did not reach statistical significance and should be regarded as non-significant tendency only. The numerically higher dissatisfaction in the hormonal group may be driven by side effects, costs, or the hormone therapy not achieving the desired effectiveness [26].

The high rates of overall treatment satisfaction in both groups indicate that perceived benefit extends beyond isolated symptom changes and may reflect broader improvements in daily functioning and well-being. In addition to surgical and medical treatment of endometriosis, due to the complexity of the condition, a multimodal therapy concept should be developed in consultation with the patient, complemented by, for example, dietary changes, osteopathy, psychotherapy, acupuncture, etc. [14, 17, 19].

## Strengths and limitations

Strengths of this study include the direct comparison of surgical and hormonal management within a single tertiary endometriosis centre, the use of patient-reported outcomes across multiple symptom domains, and the inclusion of functional and quality-of-life measures beyond pain intensity alone.

At the same time, several methodological limitations should be emphasised. The retrospective design and the use of a postal questionnaire introduce recall bias, especially because the follow-up interval between treatment and questionnaire varied and was not systemically collected and should be considered when interpreting the results. The study-specific questionnaire was designed to capture RVE-relevant domains, but it was not a validated endometriosis-specific instrument; while this allowed tailored data collection, it limits comparability with studies using standardised tools. Categorising outcomes as improved, unchanged or worsened increases interpretability but reduces statistical sensitivity and may have masked smaller treatment differences. The hormonal group was substantially smaller than the surgical group, limiting statistical power. Finally, no multivariable adjustments were performed, so confounding by indication likely influenced treatment allocation and may have attenuated or exaggerated between-group differences. Additionally, a large number of outcomes were analysed without correction for multiple testing. While this is consistent with the exploratory nature of the study, it increases the risk of type I error, and all findings should be interpreted accordingly as hypothesis-generating.

## Conclusion

In women with RVE treated in a tertiary referral centre, both surgical and hormonal treatments were associated with substantial improvements in pain and functional symptoms. Direct between-group comparisons revealed no statistically significant differences in patient-reported outcome trajectories, with consistently small effect sizes.

These findings do not demonstrate the superiority of either approach and are consistent with the possibility that hormonal therapy may represent a meaningful management option in selected patients with RVE. Given the exploratory and non-randomised design, these conclusions remain tentative.

## Supplementary Information

Below is the link to the electronic supplementary material.

Supplementary file1 (PDF 442 KB)

## Data Availability

The data that support the findings of this study are available from the corresponding author, SM, upon reasonable request.
