# Supplementary material for: Pain and functional outcomes after surgical versus hormonal treatment in rectovaginal endometriosis: a retrospective cohort study
Source: Arch Gynecol Obstet. 2026 Jun 21;313(1):208. doi: 10.1007/s00404-026-08495-z (PMC13284012; doi:10.1007/s00404-026-08495-z)
Supplement: Supplementary file 1 — Supplementary file1 (PDF 442 KB) [file 404_2026_8495_MOESM1_ESM.pdf]

**Allgemeine Fragen:**	**Erste Regel mit Jahren**<br>**Kinderwunsch:** erfüllt / unerfüllt / keiner / prospektiv<br>**Schwangerschaften:** wie viele?<br>**Geburten:** wie viele ?<br>Besonderheiten?
**Diagnose Endometriose**	seit wann:
**Rektovaginale Endometriose**	seit wann:
**Sonstige Erkrankungen**	
**Beschwerden VOR der Therapie**<br>Bitte tragen sie in der visuellen Analogskala ihre typische Schmerzstärke ein	**Unterbauchschmerzen:** Ja / Nein seit:<br>zyklusabhängig: Ja / Nein seit:<br>0 1 2 3 4 5 6 7 8 9 10<br>0 = Keine Schmerzen 10 = stärkster vorstellbarer Schmerz<br><br>**Schmerzhafte Menstruation:** Ja / Nein seit:<br>0 1 2 3 4 5 6 7 8 9 10<br>0 = Keine Schmerzen 10 = stärkster vorstellbarer Schmerz<br>**Partnerschaft:** Ja / Nein seit:<br>**Schmerzen beim Geschlechtsverkehr:** Ja / Nein seit:<br>0 1 2 3 4 5 6 7 8 9 10<br>0 = Keine Schmerzen 10 = stärkster vorstellbarer Schmerz<br>**Schmerzen beim Wasserlassen:** Ja / Nein seit:<br>0 1 2 3 4 5 6 7 8 9 10<br>0 = Keine Schmerzen 10 = stärkster vorstellbarer Schmerz<br>**Schmerzen beim Stuhlgang:** Ja / Nein seit:<br>0 1 2 3 4 5 6 7 8 9 10<br>0 = Keine Schmerzen 10 = stärkster vorstellbarer Schmerz<br><br>**Fragen zur Verdauung:**<br>Verstopfung / Durchfall / Verstopfung-Durchfall im Wechsel / Unauffällig<br>Wie häufig hatten sie Stuhlgang: x pro Tag; x pro Woche<br>Mussten sie beim Stuhlgang stark pressen? nein / selten / häufig / oft<br>Benötigten sie Abführmittel: nein / selten / häufig / oft<br>Blähung: nein / selten / häufig / oft<br>Blut im Stuhl: Ja / Nein, wenn ja: während der Menstruation Ja / Nein<br><br>**Fragen zur Blasenfunktion:**<br>Wie häufig müssten sie Wasser lassen: x pro Tag<br>Mussten sie dabei stark pressen? nein / selten / häufig / oft<br>Hatten sie das Gefühl, dass die Blasenentleerung unvollständig war?<br>nein / selten / häufig / oft<br>Wurden Restharnmengen nach Blasenentleerung in der Blase festgestellt?<br>nein / selten / häufig / oft wenn ja: wie viele ml?<br>War ihr Harnstrahl normal / schwach / verlangsamt / verlängert<br>Wurde ein Harnstau (Rückstau von Urin in die Niere) festgestellt?<br>Ja / nein

**Schmerzmittelbedarf**	Ja, welche / Nein
**Sind Sie wegen Endometriose operiert worden?**<br><br>**Wurde die rektovaginale Endometriose entfernt, wenn ja , wie**	wie oft, wann, was wurde gemacht?<br><br>Ja nein
**Wird oder wurde eine hormonelle Therapie durchgeführt?**	z.B. die Pille, Gestagenmonopräparate, Hormonspirale?<br>Was und seit wann:
**Sonstige Therapien**	
**Beschwerden nach der Therapie**<br><br>Bitte tragen sie in der visuellen Analogskala ihre typische Schmerzstärke ein	**Unterbauchschmerzen:** Ja / Nein seit:<br>zyklusabhängig: Ja / Nein seit:<br>0 1 2 3 4 5 6 7 8 9 10<br>0 = Keine Schmerzen 10 = stärkster vorstellbarer Schmerz<br><br>**Schmerzhafte Menstruation:** Ja / Nein seit:<br>0 1 2 3 4 5 6 7 8 9 10<br>0 = Keine Schmerzen 10 = stärkster vorstellbarer Schmerz<br>**Partnerschaft:** Ja / Nein seit:<br>**Schmerzen beim Geschlechtsverkehr:** Ja / Nein seit:<br>0 1 2 3 4 5 6 7 8 9 10<br>0 = Keine Schmerzen 10 = stärkster vorstellbarer Schmerz<br>**Schmerzen beim Wasserlassen:** Ja / Nein seit:<br>0 1 2 3 4 5 6 7 8 9 10<br>0 = Keine Schmerzen 10 = stärkster vorstellbarer Schmerz<br>**Schmerzen beim Stuhlgang:** Ja / Nein seit:<br>0 1 2 3 4 5 6 7 8 9 10<br>0 = Keine Schmerzen 10 = stärkster vorstellbarer Schmerz

	**Fragen zur Verdauung:**<br>Verstopfung / Durchfall / Verstopfung-Durchfall im Wechsel / Unauffällig<br>Wie häufig haben sie Stuhlgang: x pro Tag; x pro Woche<br>Müssen sie beim Stuhlgang stark pressen? nein / selten / häufig / oft<br>Benötigen sie Abführmittel: nein / selten / häufig / oft<br>Blähung: nein / selten / häufig / oft<br>Blut im Stuhl: Ja / Nein, wenn ja: während der Menstruation Ja / Nein<br><br>**Fragen zur Blasenfunktion:**<br>Wie häufig müssen sie Wasser lassen: x pro Tag<br>Müssen sie dabei stark pressen? nein / selten / häufig / oft<br>Haben sie das Gefühl, dass die Blasenentleerung unvollständig ist?<br>nein / selten / häufig / oft<br>Wurden Restharnmengen nach Blasenentleerung in der Blase festgestellt?<br>nein / selten / häufig / oft wenn ja: wie viele ml?<br>Ist ihr Harnstrahl normal / schwach / verlangsamt / verlängert<br>Ist ein Harnstau bekannt Ja / nein
**Schmerzmittelbedarf**	Ja, welche / Nein
**Besserung der Beschwerden?**	**Nach der Operation:** Ja / Nein<br>wie lange anhaltend?<br>**Nach der hormonellen Therapie:** Ja / Nein<br>wie lange anhaltend?<br>**Nach der Operation und hormonelle Therapie:** Ja / Nein<br>wie lange anhaltend?<br>**Nach sonstiger Therapie:** Ja / Nein<br>wie lange anhaltend
**Lebensqualität vor der Therapie**<br>**Lebensqualität nach der Therapie**	Sehr eingeschränkt, eingeschränkt, wenig eingeschränkt, nicht eingeschränkt<br><br>Sehr eingeschränkt, eingeschränkt, wenig eingeschränkt, nicht eingeschränkt
**Postoperative Komplikationen**	Keine
	Harnblasenfunktion: eingeschränkt / uneingeschränkt, Besserung nach OP: ja / nein<br>Blasenentleerungsstörungen ja / nein, Besserung nach OP: ja /nein
	Darmfunktion: eingeschränkt / uneingeschränkt, Besserung nach OP: ja / nein<br>Darmfunktionsstörung? Ja / nein
	Geschlechtsverkehr: eingeschränkt / uneingeschränkt, Besserung nach OP: ja / nein
	Sonstige: z. B. Wundheilungsstörungen?

**Sind Sie mit Ihrer Entscheidung für eine Operation rückblickend zufrieden? Bitte teilen sie uns ihre Erfahrung mit.**	Sehr zufrieden / zufrieden / zufrieden stellend / unzufrieden / sehr unzufrieden<br>Ich bereue die Entscheidung zur OP nicht, weil:<br><br>Ich bereue die Entscheidung zur OP, weil:

Datum
